# Abnormal X chromosome inactivation and sex-specific gene dysregulation after ablation of FBXL10

**DOI:** 10.1186/s13072-016-0069-1

**Published:** 2016-05-31

**Authors:** Mathieu Boulard, John R. Edwards, Timothy H. Bestor

**Affiliations:** Department of Genetics and Development, College of Physicians and Surgeons of Columbia University, 701 W. 168th St., New York, NY 10032 USA; Center for Pharmacogenomics, Department of Medicine, Washington University School of Medicine, St. Louis, MO 63110 USA

**Keywords:** Xist, Sexual dimorphism, CpG-rich promoters, FBXL10, Histone demethylase, X inactivation

## Abstract

**Background:**

Almost all CpG-rich promoters in the mammalian genome are bound by the multidomain FBXL10 protein (also known as KDM2B, JHDM1B, CXXC2, and NDY1). FBXL10 is expressed as two isoforms: FBXL10-1, a longer form that contains an N-terminal histone demethylase domain with C-terminal F-box, CXXC, PHD, RING, and leucine-rich repeat domains, and FBXL10-2, a shorter form that initiates at an alternative internal exon and which lacks the histone demethylase domain but retains all other annotated domains. Selective deletion of *Fbxl10*-*1* had been reported to produce a low penetrance and variable phenotype; most of the mutant animals were essentially normal. We constructed mutant mouse strains that were either null for *Fbxl10*-*2* but wild type for *Fbxl10*-*1* or null for both *Fbxl10*-*1* and *Fbxl10*-*2*.

**Results:**

Deletion of *Fbxl10*-*2* (in a manner that does not perturb expression of *Fbxl10*-*1*) produced a phenotype very different from the *Fbxl10*-*1* mutant, with craniofacial abnormalities, neural tube defects, and increased lethality, especially in females. Mutants that lacked both FBXL10-1 and FBXL10-2 showed embryonic lethality and even more extreme sexual dimorphism, with more severe gene dysregulation in mutant female embryos. X-linked genes were most severely dysregulated, and there was marked overexpression of *Xist* in mutant females although genes that encode factors that bind to Xist RNA were globally downregulated in mutant female as compared to male embryos.

**Conclusions:**

FBXL10 is the first factor shown to be required both for the normal expression and function of the *Xist* gene and for normal expression of proteins that associate with Xist RNA; it is proposed that FBXL10 coordinates the expression of Xist RNA with proteins that associate with this RNA. The function of FBXL10 is largely independent of the histone demethylase activity of the long form of the protein.

**Electronic supplementary material:**

The online version of this article (doi:10.1186/s13072-016-0069-1) contains supplementary material, which is available to authorized users.

## Background

FBXL10 is a multidomain chromosomal protein that is localized to essentially all CpG-rich promoters via a CXXC domain that binds to unmethylated CpG dinucleotides [1, 3]. FBXL10 is present in a variant form of Polycomb Repressive Complex 1 (PRC1), but most FBXL10 is deposited at promoters that are not associated with Polycomb factors. Many biological functions have been ascribed to FBXL10; among these are the regulation of nucleolar function [3], organization of Polycomb Repressive Complexes [3, 4], self-renewal of stem cells [5], and regulation of histone H2A ubiquitylation [6]. However, these studies were conducted prior to the demonstration that FBXL10 is bound to all CpG-rich promoters and none utilized mouse strains null for *Fbxl10.* FBXL10 has also been reported to be an oncogene and a tumor suppressor in leukemia [7, 8]. The biological activities of FBXL10 are usually attributed to the JmjC histone demethylase domain, although FBXL10 also contains CXXC, PHD, RING, F-box, and leucine-rich repeat (LRR) domains. It was recently reported that FBXL10 is required for the protection of sequences bound by Polycomb Repressive Complexes from *de novo* methylation, while promoters bound by FBXL10 but not by Polycomb Repressive Complexes did not undergo *de novo* methylation after removal of FBXL10 [9].

Ablation of the long form of the protein (the JmjC-containing FBXL10-1) was reported to produce a fully recessive phenotype in which most homozygous mice were of normal phenotype but some displayed coloboma, exencephaly, and rare tail kinks [10]. A single conservative amino acid substitution that affects both FBXL10-1 and FBXL10-2 causes paunch calf syndrome with craniofacial dysmorphia and perinatal lethality in Romagnolo cattle; this mutation is also fully recessive [11]. A deletion that removed the CXXC domain (which is common to both FBXL10-1 and FBXL10-2) was reported to produce increased lethality and developmental abnormalities in heterozygous mice as a result of haploinsufficiency, but this mutant allele produced large amounts of an abnormal protein that could have exerted neomorphic effects [4]. The semidominant character of this allele contrasts with the fully recessive nature of all other mutant alleles of *Fbxl10* (including alleles described here).

We report here that a mutant allele that deleted *Fbxl10*-*2* in a manner that did not affect expression of *Fbxl10*-*1* was fully recessive and produced a phenotype that differed markedly from the *Fbxl10*-*1* deletion. The *Fbxl10*-*2* mutation caused craniofacial abnormalities, with high rates of perinatal lethality and a strain-dependent cleft palate and eyes-open-at-birth phenotype. We observed that female homozygotes had a more severe phenotype and that very few survived to adulthood. We further investigated the sexual dimorphism in embryos that lacked both FBXL10-1 and FBXL10-2 and found dysregulation of many genes in female embryos, with pronounced dysregulation of X-linked genes and strong overexpression of Xist specifically in female embryos, with a net decrease in X-linked gene expression that was not detectable in male embryos. Male embryos showed greatly decreased *Tsix* expression but did not detectably reactivate *Xist*. The data indicate that removal of FBXL10 results in widespread dysregulation of gene expression, with much larger effects in female embryos and even greater dysregulation of X-linked genes. FBXL10 is a novel factor that is required for normal expression of *Xist* and *Tsix* and for the coordinated expression of Xist RNA and the protein factors that associate with this RNA.

## Methods

### Mouse strains

**Fig. 1:**
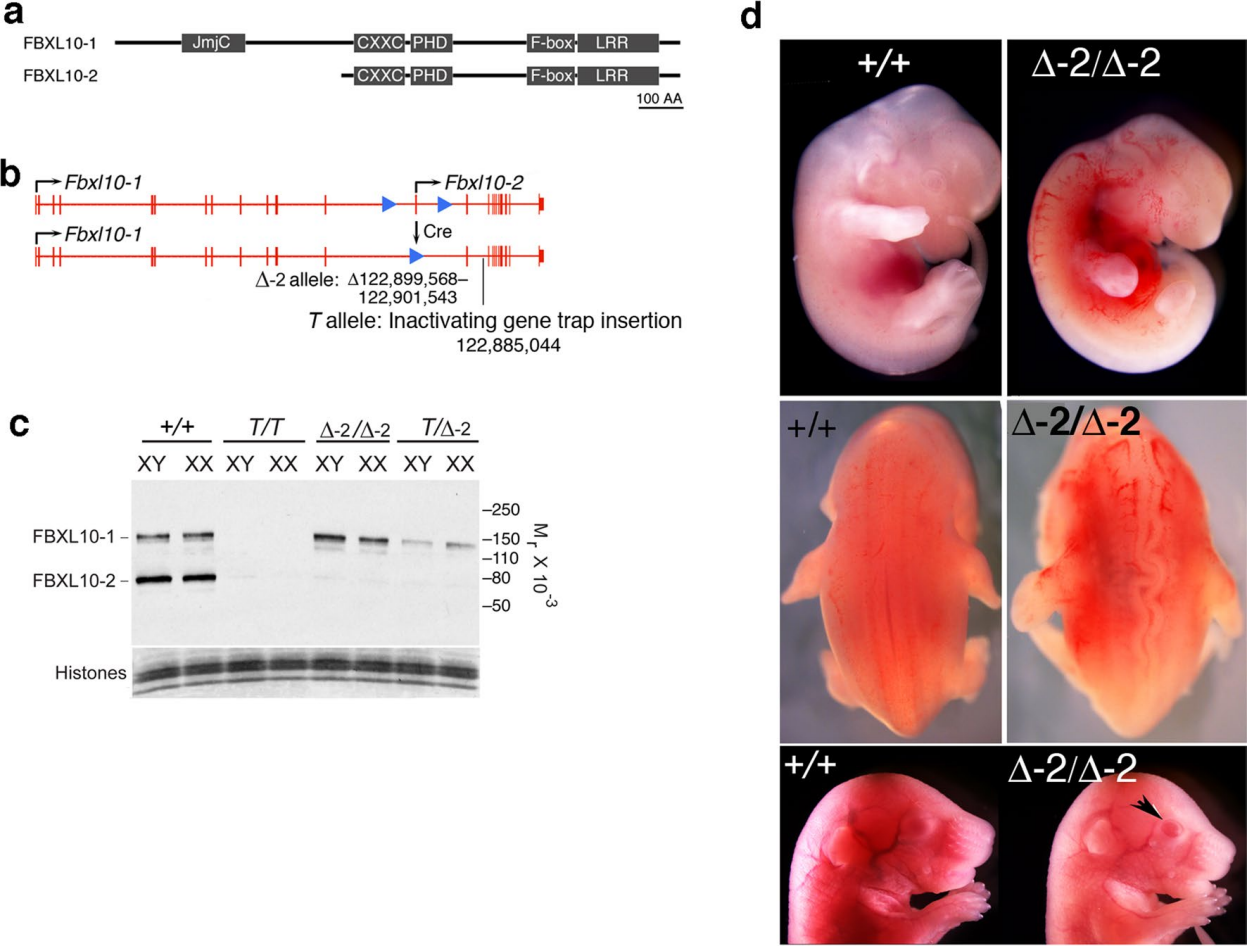
**a** Domain organization of FBXL10-1 and FBXL10-2 proteins. **b** Intron–exon structure of *Fbxl0*-*1* and *Fbxl0*-*2* genes and Cre-mediated deletion of promoter and first exon of *Fbxl10*-*2.* LoxP sites introduced by homologous recombination are shown as *blue arrowheads*. The alternative first exon of *Fbxl10*-*2* is not present in transcript of *Fbxl10*-*1*. Gene trap truncation (*T* allele) is a null allele of both *Fbxl10*-1 and *Fbxl10*-*2* and is described in [9]. **c** Immunoblot confirmation that the deletion shown in **b** and denoted as Δ-2 prevents expression of FBXL10-2. The gene trap allele denoted as *T* eliminates both forms of FBXL10. **d** Craniofacial abnormalities in E12.5 embryos (*top row*) and E17.5 embryos (*bottom row*) homozygous for the Δ-2 allele. Compression of the anterior portion of the head is apparent (*top* and *bottom rows*), as is the eyes-open-at-birth phenotype (*black arrowhead at bottom*). A tortuous neural tube was observed in both *T/T* and Δ-2*/*Δ-2 embryos (*middle row*)

All animal experimentation was conducted under protocols approved by the Institutional Animal Care and Use Committee of Columbia University. The *Fbxl10*^*T/T*^ null strain was described in [9], and construction of the *Fbxl10*^∆−2/∆−2^ strain in which *Fbxl10*-*2* is selectively deleted is diagrammed in Fig. 1a and Additional file 1: Fig. S1. All PCR primer sequences and other information related to the construction of this strain are available on request.

### RNA-seq analysis

Two biological replicates for each genotype of both sexes were analyzed. Total RNA was extracted from single embryos dissected at embryonic day E9.5. The sex of the embryos was determined by PCR detection of the *Sry* gene using genomic DNA extracted from the yolk sac. RNA-seq reads were mapped to the mouse genome (mm9) using TopHat (v1.4.1; [12]). Mapped reads were assigned to RefSeq annotated genes using htseq-count (version 0.5.3p9; http://www-huber.embl.de/users/anders/HTSeq/doc/count.html). Differential expression was assessed by edgeR (http://www.ncbi.nlm.nih.gov/pmc/articles/PMC2796818/) with TMM normalization. Log2 fold-changes were computed after applying a floor of 1 cpm to ensure that genes with very low expression but large fold-changes did not affect the analysis. Pearson’s correlation coefficient was used to compare the global variation in expression levels in +/+ and *T*/*T* mice. Distributions of log2 fold expression changes between autosomal and ChrX genes were compared using the Mann–Whitney *U* test.

### GSEA analysis

Gene set enrichment analysis was performed as in [13] using the log2 fold-change to rank genes. A custom gene signature from 81 Xist interacting factors [14] was used for the analysis. The density of Xist bound factors was computed as the number of genes in a 2000 gene window normalized by the window size as the window slides across the ranked gene list. P values are those reported from GSEA for the enrichment of Xist bound factors.

### ChIP-Seq analysis

FBXL10 ChIP-Seq data from mouse ES cells are from GSE41316 [3].

### Data access

The RNA-seq data were deposited at the Gene Expression Omnibus under the Accession number GSE76552.

## Results

### Selective deletion of FBXL10-2: sexually dimorphic phenotype distinct from FBXL10-1 mutation

As shown in Fig. 1a, b, FBXL10-2 differs from FBXL10-1 by the absence of an N-terminal domain that contains a JmjC histone demethylase domain. As mentioned previously, a deletion that selectively removed FBXL10-1 was reported to have a weakly penetrant recessive phenotype characterized by low-frequency exencephaly, coloboma, and tail kinks, although most homozygous mutants were without visible phenotype [10]. We selectively removed FBXL10-2 by introduction of loxP sites followed by Cre-mediated excision of the alternative internal promoter and first exon that drive expression of FBXL10-2 (Fig. 1b, Additional file 1: Fig. S1). As shown in Fig. 1b, c, the deletion removed all detectable FBXL10-2 protein without measurable effect on FBXL10-1.

The FBXL10-1 and FBXL10-2 deletions had markedly different phenotypes. The FBXL10-2 deletion did not show exencephaly, coloboma, or kinked tail as did the FBXL10-1 mutants. In contrast, surviving animals lacking FBXL10-2 were runted and displayed craniofacial abnormalities (Additional file 1: Fig. S2a), while mutants that died prenatally showed craniofacial and neural tube abnormalities (Fig. 1d). When backcrossed onto the FVB genetic background, the *Fbxl10*^∆−2/∆−2^ mutation additionally caused cleft pallet and open eyelids at birth (Fig. 1d, Additional file 1: Fig. S2b). We performed all gene expression experiments on the C57Bl6 strain background.

While *Fbxl10*-*2* is expressed at the same level in males and females (Fig. 1c), fewer females (*P* = 0.000319) than males (*P* = 0.7829) survived to postnatal day 21 (Additional file 2: Table S1).

### Abnormal development and gene expression in Fbxl10-null embryos

**Fig. 2:**
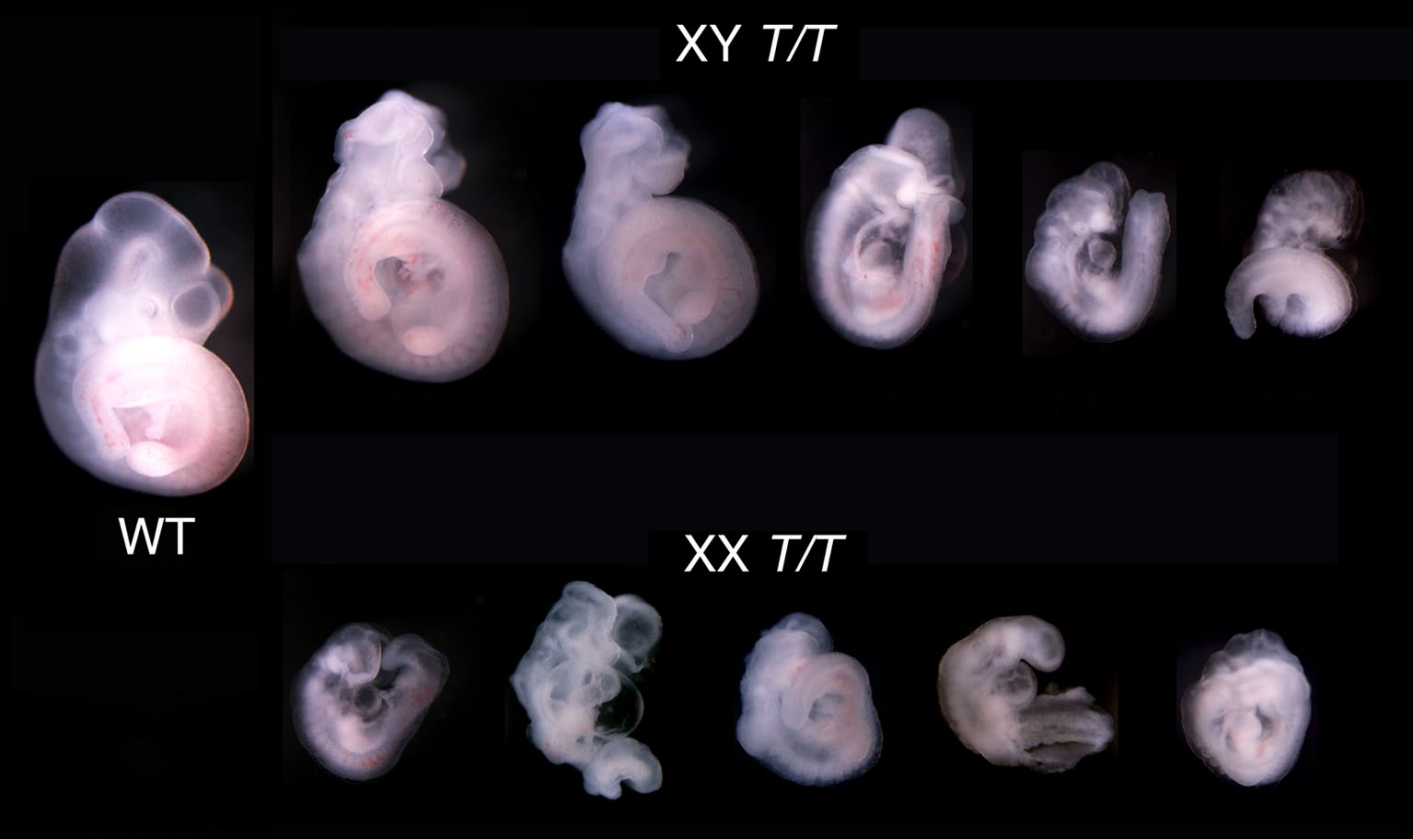
Sexually dimorphic anatomical phenotypes in *Fbxl10* ^*T/T*^ embryos. Embryos were collected at E10.5 and sexes determined by the presence or absence of the *Sry* gene. The most severely affected male embryos resembled the least severely affected female embryos

Further investigation of the sexually dimorphic phenotype was conducted in embryos homozygous for a truncating gene trap allele (*Fbxl10*^*T*^) that removes all detectable FBXL10-1 and FBXL10-2 protein and is a null allele (Fig. 1b, c). These embryos showed a much more severe phenotype and more pronounced sexual dimorphism than *Fbxl10*^∆−2/∆−2^ mutants (Fig. 1d), as shown in Fig. 2. Embryonic development ceased prior to E10.5, with multiple and severe developmental abnormalities, including neural tube kinks similar to those observed in *Fbxl10*^∆−2/∆−2^ mutants. Figure 2 shows the sexual dimorphism resulting from FBXL10-1 and FBXL10-2 ablation; the most advanced female embryos were observed to cease development at roughly the same stage at the least advanced male embryos.

**Fig. 3:**
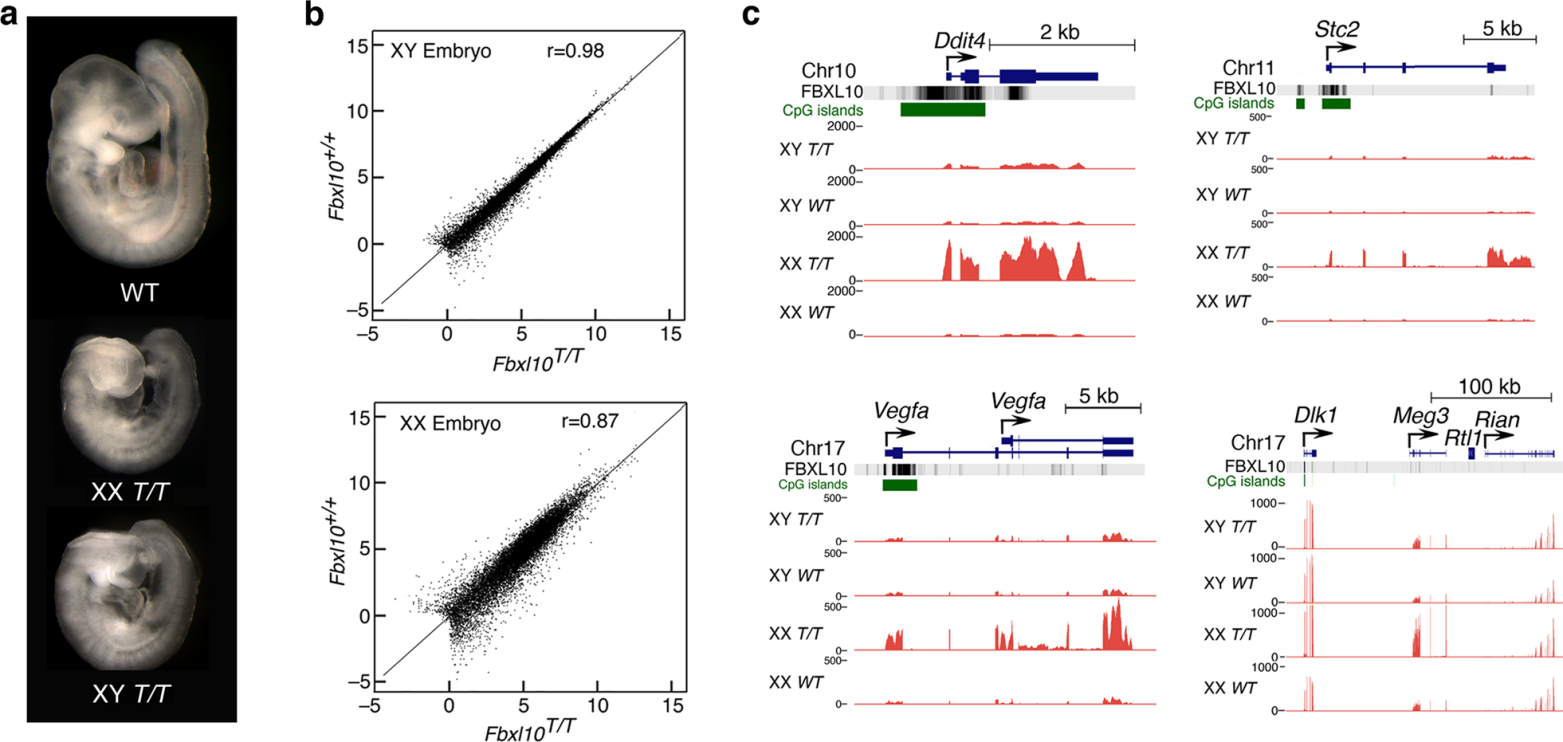
Sex-specific gene dysregulation in *Fbxl10* ^*T/T*^ embryos. **a** Representative littermate embryos dissected at embryonic day 9.5. Males and females are indistinguishable on the basis of their morphology at this stage of development. **b** Greater variation in gene expression in female mutant embryos as determined by RNA-seq. **c** Examples of sex-specific expression abnormalities in *Fbxl10* ^*T/T*^ embryos. The *Ddit4*, *Vegfa*, and *Stc2* genes, and the imprinted gene *Meg3*, are all markedly overexpressed in female *Fbxl10* ^*T/T*^ embryos

RNA-seq was performed on RNA from stage-matched female (XX) and male (XY) *Fbxl10*^*T/T*^ mutant embryos at E9.5; as shown in Fig. 3a, developmental abnormalities were much less severe at E9.5 than in the E10.5 embryos shown in Fig. 2. In agreement with the deposition of FBXL10 at all CpG-rich promoters [1, 2], its removal results in a highly complex transcriptional phenotype (Fig. 3b, c). As shown in Fig. 3b, female embryos showed more extensive dysregulation of gene expression than did male embryos, with many genes upregulated or downregulated.

Additional file 3: Table S2 shows differential gene expression in male versus female *Fbxl10* null mutants, and Fig. 3c shows examples of genes that showed sex-specific dysregulation. The imprinted *Meg3* and *Gtl2* genes [15] were notably upregulated in mutant XX embryos relative to XY embryos, although other nearby genes such as *Rian* were expressed at normal levels (Fig. 3c). Expression of *Meg3* and *Gtl2* was increased by a factor of greater than 2, which suggests that activation of the normally silent imprinted alleles was unlikely to be responsible for the increased expression. Abnormalities of gene expression were widespread and more apparent in female than in male embryos.

### Abnormal expression of X-linked genes in the absence of FBXL10

**Fig. 4:**
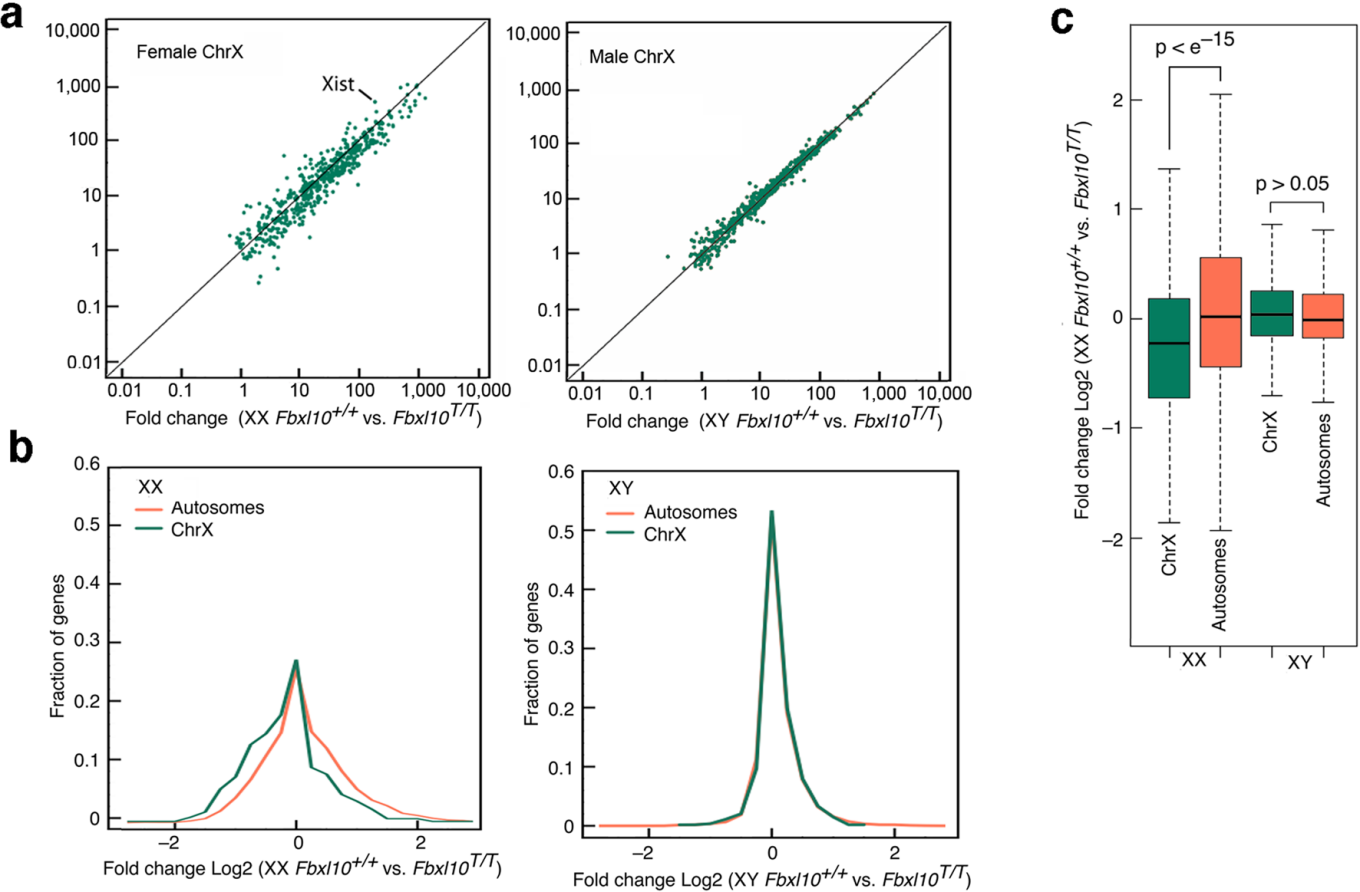
Dysregulation of X-linked genes in female but not male *Fbxl10* ^*T/T*^ embryos. **a** Transcripts from X-linked genes were computationally isolated from RNA-seq data and expression levels compared to E9.5 *Fbxl10* ^*T/T*^ versus *Fbxl10* ^+*/*+^ embryos. **b** Histogram analysis of the RNA-seq data in **a**. Both autosomal and X-linked genes are dysregulated in female *Fbxl10* ^*T/T*^ embryos, but X-linked genes are more severely affected and a greater extent of downregulation is apparent. **c** Downregulation of X-linked genes in female *Fbxl10* ^*T/T*^ embryos is of high statistical significance

RNA transcripts derived from X-linked genes were identified in RNA-seq data and the extent of dysregulation compared to that of autosomal genes in male and female *Fbxl10*-null embryos. As shown in Fig. 4a, c, a highly significant dysregulation of X-linked genes was apparent in female but not male embryos, with an overall net reduction of expression. This is apparent in the histogram of Fig. 4b, where autosomal genes can be seen to be also dysregulated to a much greater extent in female than in male embryos. Overall abnormalities of gene expression were much greater in female embryos, and dysregulation of X-linked genes (with the exception of *Tsix*; see below) was apparent only in female embryos. Quantitative RT-PCR confirmed the female-specific downregulation of ten X-linked genes (Additional file 1: Fig. S3).

### Normal reactivation of the paternally imprinted X chromosome in Fbxl10 null mutants

The observed global downregulation of the active X chromosome could arise from the failure of the paternally imprinted X chromosome to be reactivated. A defective counting in cells that have a paternally imprinted X chromosome would cause the inactivation of two X chromosomes in half the cells (the cells that randomly inactivate the X chromosome of maternal origin). We reasoned that a failure to reactivate the paternally imprinted X chromosome would lead to a skewed X inactivation pattern. We tested for potential allelic imbalance of two X-linked transcripts using single nucleotide polymorphisms (SNPs) to trace the parental origin of the transcripts (Additional file 1: Fig. S4). *Haus7* and *Sh3bgr1* are X-linked genes with reduced expression in mutant females (Additional file 1: Fig. S4a, c). Our SNP analysis showed that transcripts from each parental allele were equally represented in female mutant embryos (Additional file 1: Fig. S4b, d). Therefore, X inactivation is not skewed in female embryos that lack FBXL10, which indicates that choice in somatic X inactivation is normal in the absence of FBXL10.

### Abnormal expression of Xist RNA and Xist RNA-binding factors in Fbxl10-null female embryos

**Fig. 5:**
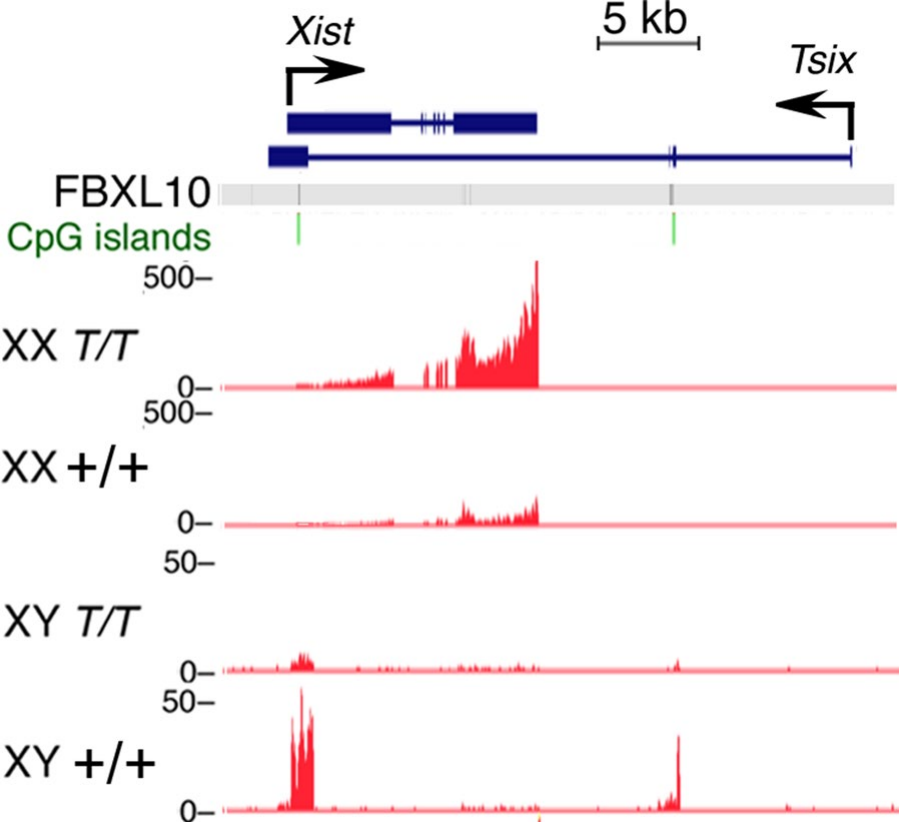
Overexpression of Xist RNA in female *Fbxl10* ^*T/T*^ embryos (*upper two rows*); underexpression of Tsix and lack of activation of Xist in male *Fbxl10* ^*T/T*^ embryos (*lower two rows*)

The *Xist* gene was found to be greatly upregulated in *Fbxl10* female mutant embryos, while the male-specific *Tsix* gene was downregulated in mutant male embryos (Fig. 5). *Xist* expression was increased by considerably more than twofold. Furthermore, no Xist transcripts were detectable in mutant male embryos, which indicates that removal of FBXL10 did not directly activate *Xist* expression.

**Fig. 6:**
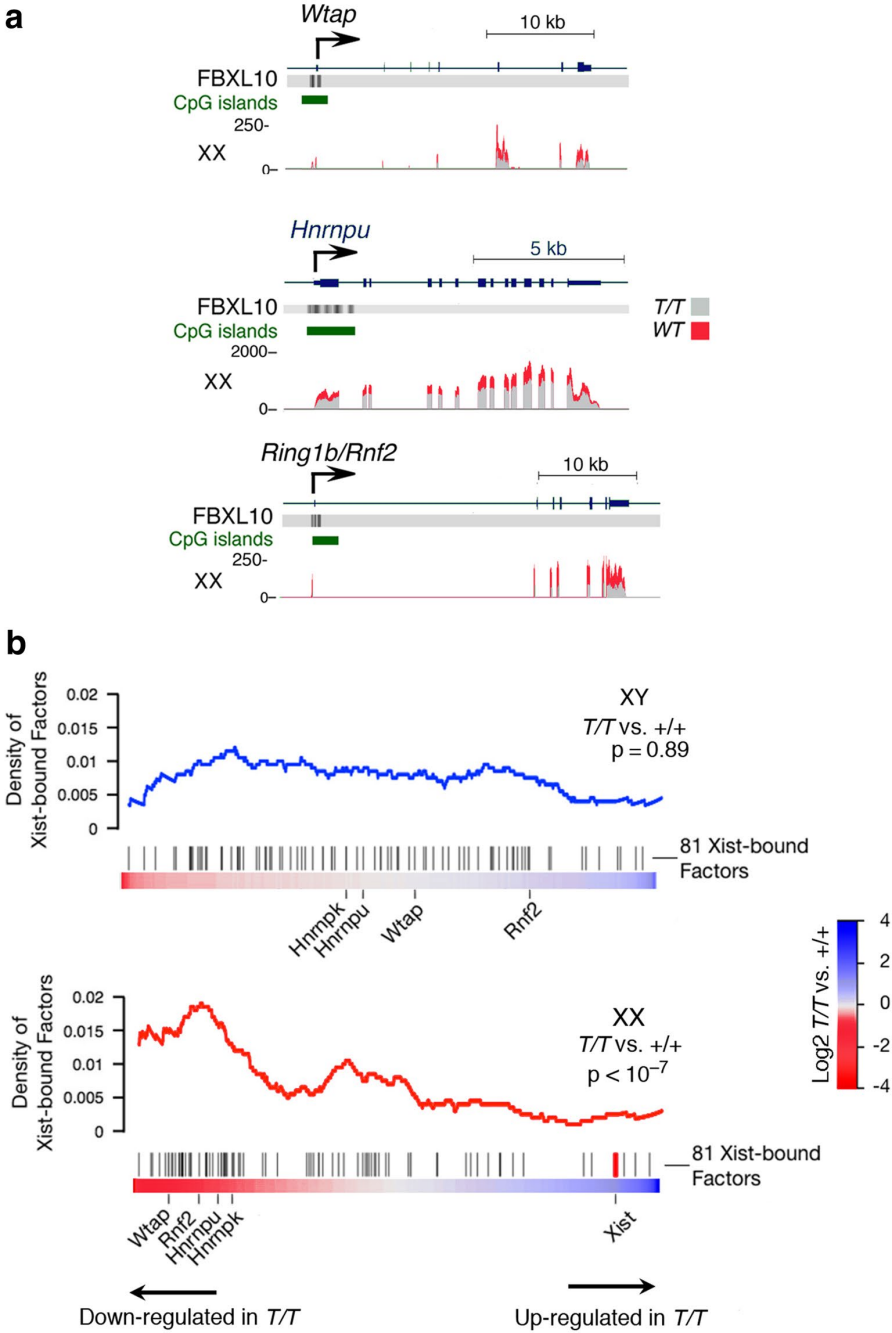
**a** Underexpression in *Fbxl10* ^*T/T*^ female embryos of selected genes that encode factors reported to interact with Xist RNA [14]. **b** Expression of genes that encode Xist RNA-binding factors in female *Fbxl10* ^*T/T*^ embryos (*bottom*); normal expression of the same genes in male *Fbxl10* ^*T/T*^ embryos (*top*). Analysis was performed via GSEA [13]. These data indicate that the overexpression of Xist RNA as shown in Fig. 5 is concomitant with reduced levels of proteins that associate with Xist RNA

A set of 81 proteins has recently been reported to bind to Xist RNA specifically in female cells that have undergone X inactivation [14]. Two of these Xist-associated proteins (RING1B/RNF2 and RYBP) have been reported to physically interact with FBXL10 [1, 6, 14]. We used statistically rigorous gene set enrichment analysis (GSEA; [13]) to evaluate the relative levels of the mRNAs that encode these 81 factors in mutant female versus mutant male embryos; the results are shown in Fig. 6. Pronounced sex-specific reductions in most of these factors were observed, together with a strong overexpression of Xist (Fig. 6b). Chu et al. [14] identified the factors WTAP, RING1B/RNF2, HNRNPK, and HNRNPU as directly involved in Xist-mediated X inactivation, and all of these factors were markedly downregulated in female but not male *Fbxl10*-null embryos (Fig. 6a).

## Discussion

Individual ablations of FBXL10-1 and FBXL-2 produced non-overlapping phenotypes. The long form deletion produced low-penetrance exencephaly, colobomo, and rare tail kinks with low mortality [10], while deletion of the short form caused high-penetrance anterior–posterior craniofacial contraction with strain-dependent cleft palate and an eyes-open-at-birth phenotype, with high perinatal lethality. This is an unusual case in which selective deletion of two isoforms of the same gene gives rise to highly dissimilar phenotypes. The data also indicate that histone demethylation via the JmjC domain of FBXL10-1 is largely dispensable for the biological activity of the protein. There are other cases in which the histone modifying domains of chromosomal proteins are largely dispensable; the histone demethylase activity of KDM6B is dispensable for the biological function of the protein [16], as is the E3 ubiquitin ligase activity of RING1B/RNF2 [17].

We observed sexual dimorphism in the form of more severe developmental defects in female mice that lacked FBXL10-2; we then tested for sexually dimorphic phenotypes in mouse embryos that lacked both the FBXL10-1 and FBXL10-2. The *Fbxl0* null mutation was found to cause much more severe developmental abnormalities and earlier death in female embryos; at E10.5, the most advanced female embryos resembled the least advanced male embryos. Analysis of gene expression abnormalities by RNA-seq on single E9.5 embryos showed much greater dysregulation of gene expression in female embryos. This was unexpected given that FBXL10 is localized to nearly all CpG-rich promoters [1, 3], which account for ~76 % of all promoters.

The sexually dimorphic phenotypes were apparent well prior to the onset of sexual differentiation, which suggested that abnormalities of sex chromosome function might be involved. Inspection of RNA-seq data showed that *Xist* was markedly overexpressed in *Fbxl10* null mutant female embryos, while *Tsix* was reduced in mutant male embryos. Deficiencies of the MYST1-(MOF)-containing MSL and NSL complexes have been reported to cause downregulation of *Tsix* and biallelic expression of *Xist* with Xist RNA coating of both X chromosomes [18], but examination of RNA-seq data showed that all of the ~20 components of the MSL and NSL complexes were expressed normally in male and female *Fbxl10* mutants (data not shown). Deletion of *Tsix* has also been reported to cause biallelic expression of *Xist* in female cells [19], but the marked downregulation of *Tsix* in male *Fbxl10* mutant embryos or ES cells did not result in detectable expression of *Xist* from the male X chromosome, and expression of *Tsix* was not detectable in mutant female embryos. Furthermore, FBXL10 is not detectably enriched at the promoters of the *Xist* or *Tsix* genes [1].

A recent study identified a set of ~81 proteins that interact with Xist RNA [14]. We found that many of these factors were expressed at reduced levels in *Fbxl10*-null female embryos but were expressed at normal levels in mutant male embryos. *Hnrnpu*, which is required for the association of the Xist complex with the inactive X chromosome [20], is reduced by more than twofold, as is the histone ubiquitin transferase RING1B/RNF2. The pronounced imbalance created by excess Xist RNA combined to the reduced concentrations of Xist binding factors will create a state in which highly heterogeneous and ectopic Xist–protein complexes in abnormally large amounts can disrupt patterns of gene expression. Genes on the X chromosome encode numerous transcription regulators; these include *Tbx22*, *Dax1*, *Sox3*, and *Pou3F4*. We suggest that ectopic Xist silencing complexes that result from the overexpression of Xist coupled to the underexpression of Xist binding factors leads to a pervasive dysregulation of gene expression driven by repression of genes on the active X chromosome. This gene dysregulation is independent of the methylation abnormalities induced by the deprivation of FBXL10, which affects only genes that are bound by Polycomb Repressive Complexes 1 and 2 [9].

**Fig. 7:**
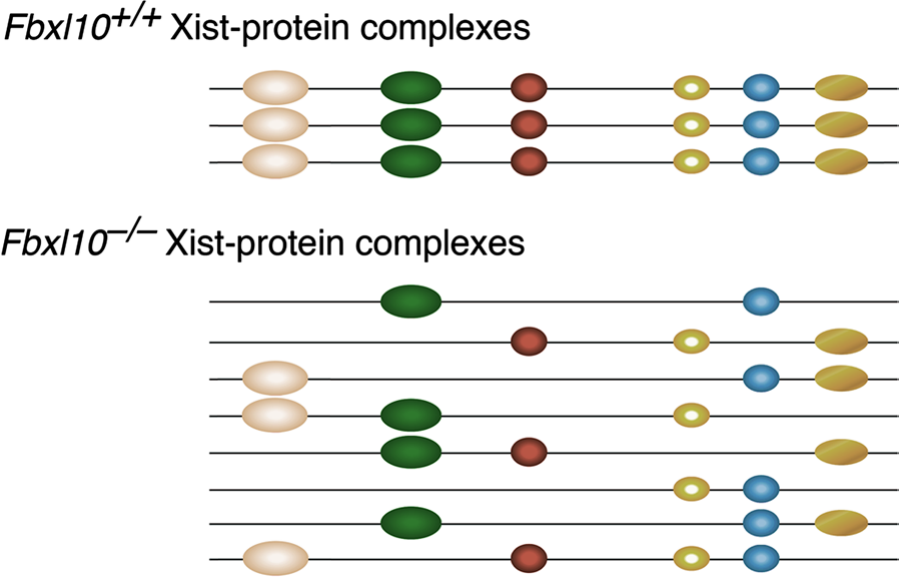
Proposed mechanism of Xist-mediated female-specific gene dysregulation in *Fbxl10* ^*T/T*^ embryos. Under this model the overexpression of Xist RNA and the underexpression of proteins that bind to Xist RNA results in the formation of increased levels of aberrant Xist RNA–protein complexes that are heterogeneous and which induce neomorphic gene dysregulation, primarily in females and largely affecting X-linked genes. Dysregulation of autosomal genes in female embryos could result from the direct effects of mistargeted abnormal Xist complexes or from the downregulation of transcription factor genes on the active X chromosome by abnormal Xist–protein complexes

We propose a model under which ectopic and heterogeneous Xist-containing silencing complexes are assembled as a result of the overexpression of Xist and underexpression of Xist binding factors after ablation of FBXL10. Under this model the ectopic, abundant, and abnormal Xist-containing silencing complexes saturate the inactive X chromosome and spill over to silence genes on the active X chromosome and possibly on autosomes after X inactivation (Fig. 7).

## Conclusions

We show that removal of FBXL10 results in overexpression of *Xist* in females, reduced expression of proteins reported to complex with Xist RNA, and greater perturbations of global gene expression profiles in females as compared to males; the data indicate that FBXL10 is a factor required for the coordinated expression of *Xist*, *Tsix*, and Xist RNA-associated factors. In the absence of FBXL10 abnormal X inactivation leads to a global reduction of X-linked gene expression and dysregulated expression of autosomal genes specifically in female embryos.

## Additional files

10.1186/s13072-016-0069-1 Description of targeting strategy used to generate the *Fbxl10* ^∆−2/∆−2^ allele, selectively deleted for *Fbxl10-2* without effect on *Fbxl10-1*. **Figure S2.** Cranial defects in *Fbxl10* ^∆−2/∆−2^ mutants. **Figure S3.** Female-specific downregulation of 10 selected X-linked genes as determined by quantitative RT-PCR. **Figure S4.** Normal allelic expression of X-linked genes indicating that downregulation of the active X chromosome is independent of its parental origin.
10.1186/s13072-016-0069-1 Female-specific mortality of *Fbxl10* ^∆−2/∆−2^ mutants. Observed frequencies of indicated genotype in males and females at birth and at 21 days after birth.
10.1186/s13072-016-0069-1 RefSeq genes with abnormal expression in females and males *Fbxl10* ^*T*/*T*^ E9.5 embryos as compared to wild type.
