# Supplementary material for: Abnormal X chromosome inactivation and sex-specific gene dysregulation after ablation of FBXL10
Source: Epigenetics Chromatin. 2016 May 31;9:22. doi: 10.1186/s13072-016-0069-1 (PMC4888662; doi:10.1186/s13072-016-0069-1)
Supplement: Supplementary file 1 — 10.1186/s13072-016-0069-1 Description of targeting strategy used to generate the Fbxl10 ∆−2/∆−2 allele, selectively deleted for Fbxl10-2 without effect on Fbxl10-1. Figure S2. Cranial defects in Fbxl10 ∆−2/∆−2 mutants. Figure S3. Female-specific downregulation of 10 selected X-linked genes as determined by quantitative RT-PCR. Figure S4. Normal allelic expression of X-linked genes indicating that downregulation of the active X chromosome is independent of its parental origin. [file 13072_2016_69_MOESM1_ESM.doc]

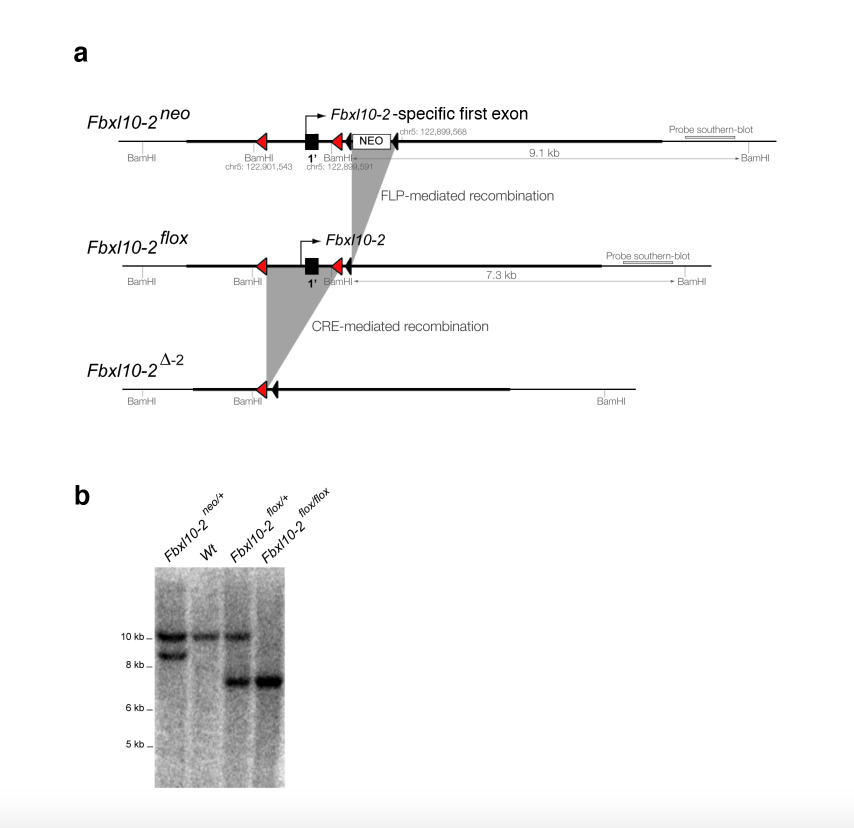


**Fig. S1.** Selective deletion of Fbxl10-2 by homologous recombination and Cre-mediated excision of the Fbxl10-2-specific exon 1. **a**., Schematic representation of the targeting strategy used to generate the *Fbxl10^∆-2/∆-2^* allele. Deletion of *Fbxl10-2*-specific promoter and first exon, which is located between exons 12 and 13 of *Fbxl10-1*.  **b**., Southern-blot confirmation of expected homologous recombination and excision events. Location of the southern blot probe is shown in **a**.


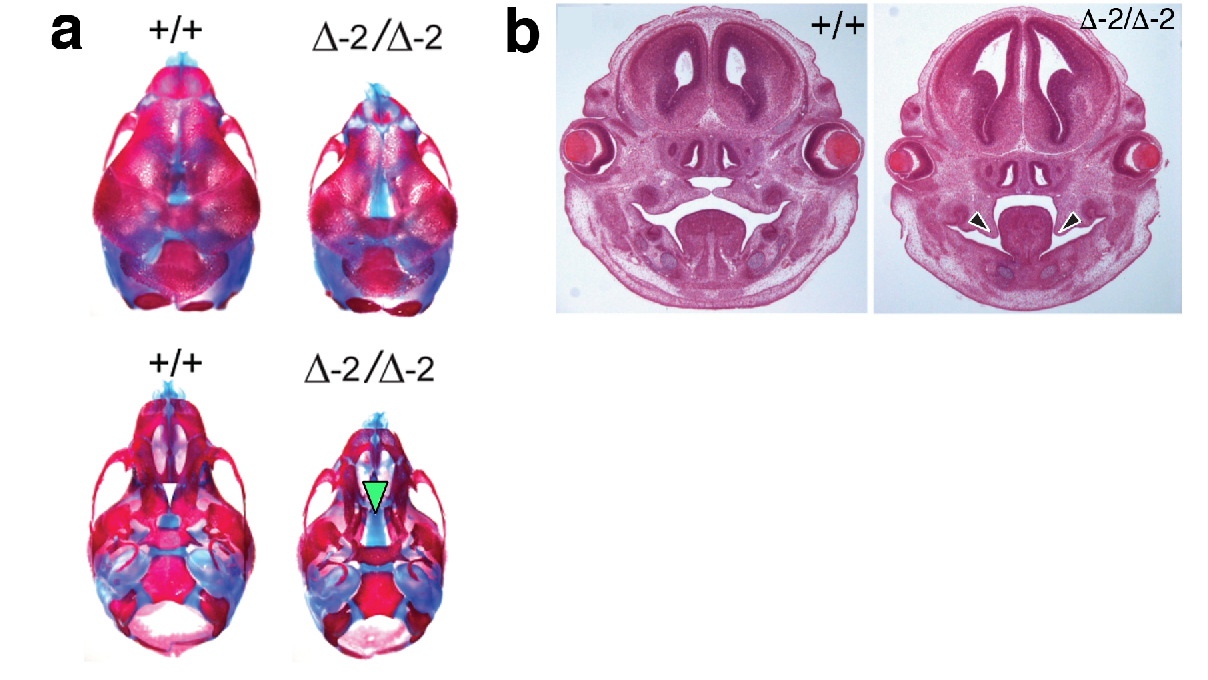


**Fig. S2.** Cranial defects in *Fbxl10^∆-2/∆-2^* mutants. **a.**, Alcian Blue/Alizarin Red skull preparations of E17.5 embryos showing the craniofacial contraction and cleft palate (green arrowhead at lower right). **b.**, Coronal sections at E14.5 showing the cleft palate (dark arrowheads).


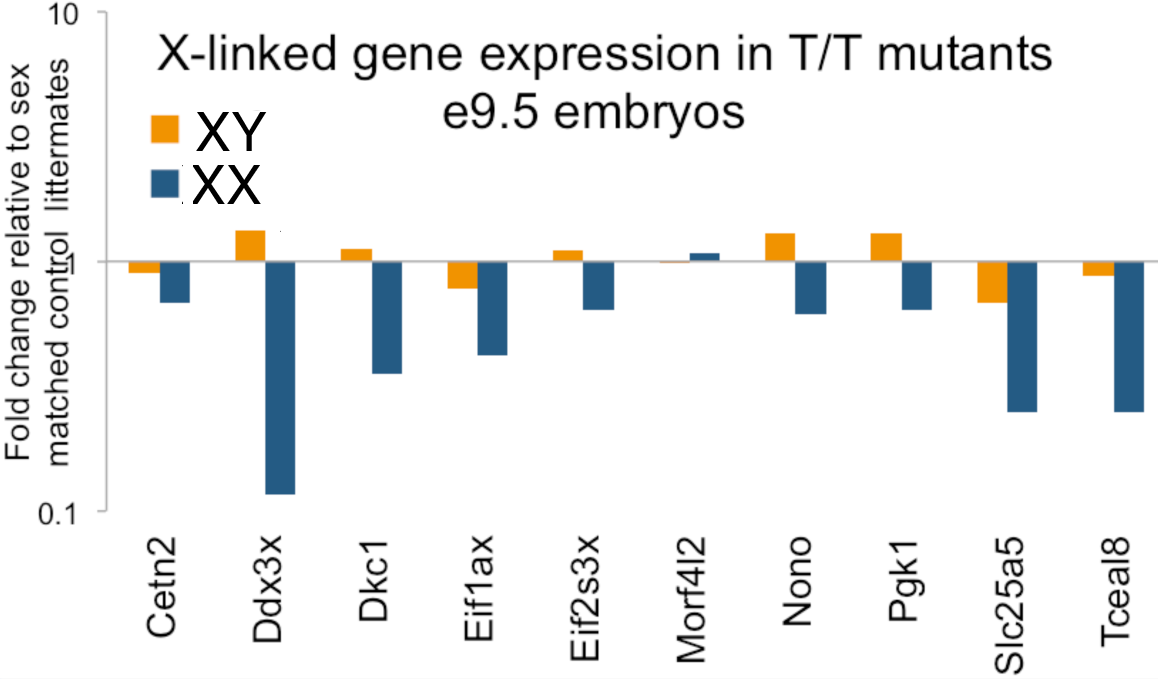


**Fig. S3.** Expression of 10 selected X-linked genes as determined by quantitative RT-PCR.


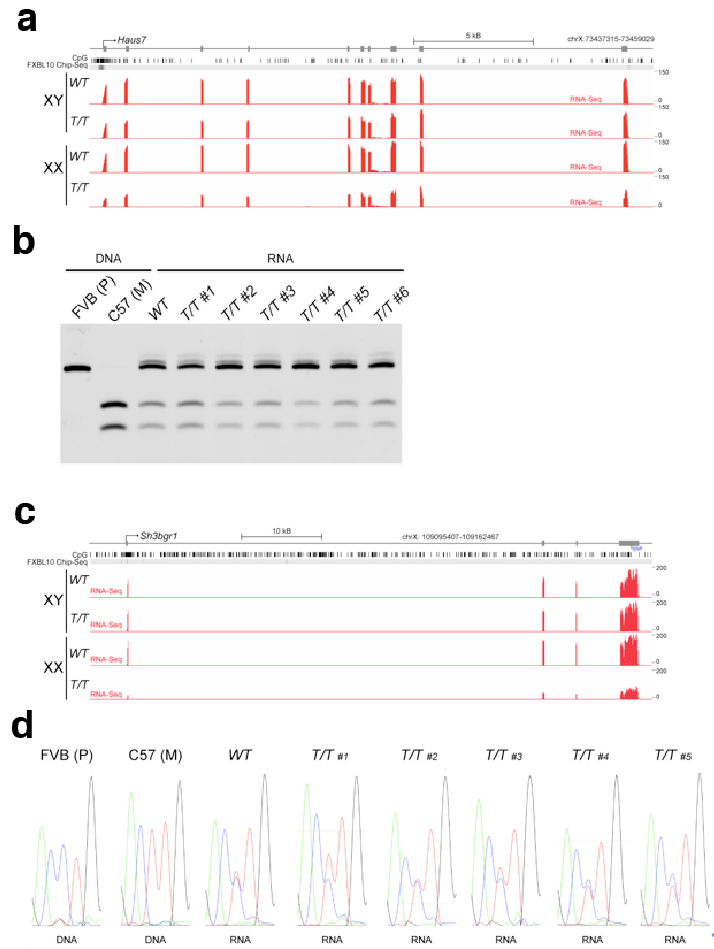
­­

**Fig. S4.** Normal allelic expression of two downregulated X-linked genes indicating that downregulation of the active X chromosome is independent of its parental origin. **a.**, Female-specific downregulation of the X-linked gene *Haus7* as measured by RNA-seq. **b.**, Allelic expression of *Haus7* in six *T/T* female embryos dissected at E9.5. Expression of the allele of maternal origin was assessed by cleavage by BstUI after PCR amplification of the cDNA. The BstUI site, located in the first exon of *Haus7*, is present in the allele of maternal origin (C57) but absent in the allele of paternal origin (FVB). **c.**, Female-specific downregulation of the X-linked gene *Sh3bgrl* as measured by RNA-seq. The SNP used to discriminate the alleles is indicated in exon 4. **d.**, Allelic expression of *Sh3bgrl* in five *T/T* female embryos dissected at E9.5 as measured by sanger sequencing of cDNA.
